# Serum myelin basic protein as a marker of brain injury in aneurysmal subarachnoid haemorrhage

**DOI:** 10.1007/s00701-019-04185-9

**Published:** 2020-01-08

**Authors:** Norbert Wąsik, Bartosz Sokół, Marcin Hołysz, Witold Mańko, Robert Juszkat, Piotr Paweł Jagodziński, Roman Jankowski

**Affiliations:** 1 Department of Neurosurgery, Poznan University of Medical Sciences, 49 Przybyszewskiego Street, 60-355 Poznan, Poland; 2 Department of Biochemistry and Molecular Biology, Poznan University of Medical Sciences, Poznan, Poland; 3 Department of Anesthesiology and Intensive Therapy, Poznan University of Medical Sciences, Poznan, Poland; 4 Department of General and Interventional Radiology, Poznan University of Medical Sciences, Poznan, Poland

**Keywords:** Aneurysmal subarachnoid haemorrhage, Outcome, Biomarker, Myelin basic protein, Endovascular coiling

## Abstract

**Background:**

Myelin basic protein (MBP) is the second most abundant protein in central nervous system myelin. Since the 1980s, it has been regarded as a marker of brain tissue injury in both trauma and disease. There have been no recent reports regarding MBP in aneurysmal subarachnoid haemorrhage (SAH).

**Methods:**

One hundred four SAH patients with ruptured aneurysms underwent endovascular treatment within 24 h of rupture, and 156 blood samples were collected: 104 on days 0–3, 32 on days 4–6 and 20 on days 9–12 post-SAH. MBP levels were assayed using ELISA and compared with the clinical status on admission, laboratory results, imaging findings and treatment outcome at 3 months.

**Results:**

MBP levels on days 0–3 post-SAH were significantly higher among poor outcome patients (*p* < 0.001), non-survivors (*p* = 0.005), patients who underwent intracranial intervention (*p* < 0.001) and patients with intracerebral haemorrhage (ICH; *p* < 0.001). On days 4–6 post-SAH, significantly higher levels were found following intracranial intervention (*p* = 0.009) and ICH (*p* = 0.039). There was clinically relevant correlation between MBP levels on days 0–3 post-SAH and 3-month Glasgow Outcome Scale (cc = − 0.42) and also ICH volume (cc = 0.48). All patients who made a full recovery had MBP levels below detection limit on days 0–3 post-SAH. Following endovascular aneurysm occlusion, there was no increase in MBP in 86 of the 104 patients investigated (83%).

**Conclusions:**

The concentration of MBP in peripheral blood after intracranial aneurysm rupture reflects the severity of the brain tissue injury (due to surgery or ICH) and correlates with the treatment outcome. Endovascular aneurysm occlusion was not followed by a rise in MBP in most cases, suggesting the safety of this technique.

## Introduction

Myelin basic protein (MBP) is the second most abundant protein in the central nervous system (CNS) myelin after the proteolipid protein. It is essential in the formation of the myelin sheath by oligodendrocytes. On the cellular level, the main role of MBP is to provide adhesion of the cytosolic surfaces of the multilayered myelin sheath; it also binds the cytoskeleton to the cell membrane and mediates extracellular signals to the cytoskeleton [1]. Since the 1980s, MBP has been regarded as a marker of brain tissue injury in trauma and disease. Elevated serum levels of MBP were observed in traumatic brain injury [2, 15], whilst elevated cerebrospinal fluid (CSF) levels were found in multiple sclerosis, benign and malignant intracranial tumours, CNS infection and cerebrovascular accidents [3, 6, 8]. Despite this, we are not aware of any recent studies focused specifically on MBP in aneurysmal subarachnoid haemorrhage (SAH). The authors have identified three studies from 1984, 1984 and 2001, all with fewer than thirty subjects whose aneurysms were managed by clipping; in these studies, MBP was measured using the radioimmunoassay method [4, 5, 12]. Following the shift of management from clipping to coiling, and the development of newer assay techniques for MBP, we feel further study of this area is justified.

## Methods and materials

### Study population and SAH management

This study was approved by the local bioethics committee and performed in accordance with the Declaration of Helsinki. One hundred four patients met the inclusion criteria for this study: (1) informed written consent from the patient or family; (2) SAH confirmed by head computerised tomography (CT); (3) aneurysm identified by digital subtraction angiography and managed endovascularly within 24 h of rupture; (4) aged over 18 years; (5) no history of neurological disease or active systemic inflammatory or neoplastic disease; (6) not pregnant. Patients were managed in the neurointensive care unit, and continuous intravenous infusion of nimodipine was carried out for at least 14 days, whilst euvolemia was maintained. Intracranial pressure (ICP) was monitored in the unconscious patients, and standard stepwise management of raised ICP was instituted, leading to decompressive craniectomy (DC; unilateral fronto-temporo-parietal craniectomy) if necessary. Acute hydrocephalus was managed with external ventricular drainage (EVD). Head CT scan was performed in all patients within 48 h of endovascular treatment and any other intracranial intervention. Further imaging and laboratory tests were applied as appropriate to the clinical situation. The clinical status on admission and functional outcome at 3 months were assessed by the neurosurgeon.

### Sample collection and assays

Peripheral blood samples were collected in all patients on days 0–3 post-SAH (also referred to as early MBP) and on days 4–6 and 9–12 post-SAH where possible. When intracranial intervention was performed, efforts were made to draw samples within 12 h. Serum-separating tubes were used to collect peripheral venous blood. The contents of the tubes were allowed to clot at 4 °C for 30–60 min. The samples were then centrifuged at 2000*g* for 10 min, and aliquots were taken immediately for storing at − 80 °C. MBP concentrations were measured using the commercially available ELISA kit (DuoSet DY4228-05, R&D Systems, Minneapolis, Minnesota, USA). The assay range is 78 to 5000 pg/ml.

### Statistical analysis

Analysis was carried out after dichotomising patients into survivors and non-survivors or into poor outcome (Glasgow Outcome Scale [GOS] score 1–3) and favourable outcome (GOS score 4–5) patients. STATISTICA 10 software (StatSoft Inc., Tulsa, OK, USA) was used to perform statistical analysis and create figures. The normality of data distribution was assessed using the Shapiro-Wilk test. The Mann-Whitney *U* test was used to compare two independent samples where there was lack of normal distribution in at least one of them. Probability value less than 0.05 was considered statistically significant. Correlation was assessed by Spearman’s rank order correlation coefficient test. Correlation coefficient > 0.4 or < − 0.4 was considered clinically relevant. The authors describe an MBP concentration above sensitivity threshold of the ELISA kit (> 78 pg/ml) as “elevated”, whilst concentrations below this level are addressed as “below detection limit” or “undetectable”. Sensitivity thresholds for MBP are marked in all figures. Results in the figures and tables are presented as median and interquartile range and base exclusively on data from patients with elevated MBP.

## Results

**Fig. 1:**
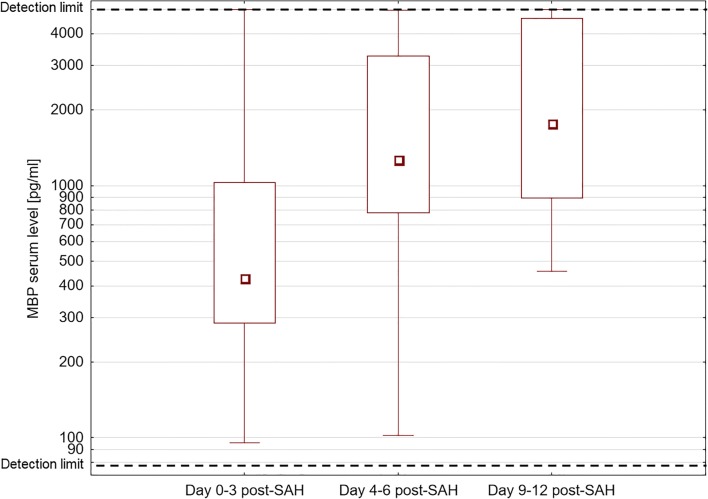
Box plot: MBP level on days 0–3, 4–6 and 9–12 post-SAH in patients with elevated MBP levels. Results are presented as median (empty square), interquartile range (box), minimum, and maximum values (whiskers); note the logarithmic scale. Dashed lines mark sensitivity thresholds of the ELISA kit

One hundred fifty-six blood samples were collected from 104 SAH patients: 104 on days 0–3 post-SAH, 32 on days 4–6 post-SAH and 20 on days 9–12 post-SAH. Elevated MBP levels were found in 18 out of 104 patients (17%) on days 0–3, 14 out of 32 on days 4–6 (44%) and 10 out of 20 on days 9–12 (50%). Table 1 presents detailed characteristics of the SAH patients with elevated serum MBP levels. Median observed values of MBP in this group on days 0–3, 4–6 and 9–12 post-SAH are presented in Fig. 1.

**Table 1:** Characteristics of the patients with elevated serum MBP levels

Male	8/18 (44%)		
Age (years)	62 (55–68)		
Aneurysm location			
Middle cerebral artery	8 (44%)		
Anterior communicating artery	3 (17%)		
Anterior cerebral artery	1 (6%)		
Basilar artery	1 (6%)		
Internal carotid artery	5 (28%)		
Aneurysm diameter [mm]	7.3 (5.0–9.6)		
Modified Fisher score	4 (4–4)		
Intracerebral hematoma	11/18 (61%)		
HH grade on admission	4 (4–5)		
WFNS grade on admission	4.5 (4–5)		
GCS on admission	6 (3–7)		
Treatment outcome according to GOS at 3 months			
5 (no/low disability)	0 (0%)		
4 (moderate disability)	1 (6%)		
3 (severe disability)	3 (17%)		
2 (persistent vegetative state)	6 (33%)		
1 (death)	8 (44%)		
	Days 0–3 post-SAH	Days 4–6 post-SAH	Days 9–12 post-SAH
MBP level (pg/ml)	430.9 (284–1025)	1269.5 (779–3255)	1769.5 (893–4593
CRP level (mg/l)	79.2 (11.7–139.6)	197.4 (114.7–289.1)	87.2 (64.1–191.0)
WBC count (10^3^/μl)	16.41 (15.4–18.9)	11.29 (9.3–11.9)	11.93 (11.2–13.1)
Hgb level (g/dl)	13.00 (11.8–14.0)	10.70 (8.9–11.6)	11.40 (10.3–11.9)
Glucose (mg/dl)	154 (140–167)	162 (129–198)	147 (140–171)
Sodium (mmol/l)	140 (139–147)	153 (141–163)	142 (139–146)
Potassium (mmol/l)	3.79 (3.6–4.0)	3.70 (3.6–4.1)	3.80 (3.6–3.9)

Values are presented as median and interquartile range or count (percentage)

*CRP* C-reactive protein, *GCS* Glasgow Coma Scale, *GOS* Glasgow Outcome Scale, *Hgb* haemoglobin, *HH* Hunt and Hess scale, *MBP* myelin basic protein, *WBC* white blood cell, *WFNS* World Federation of Neurosurgical Societies scale

### Clinical status at admission and treatment outcome

**Fig. 2:**
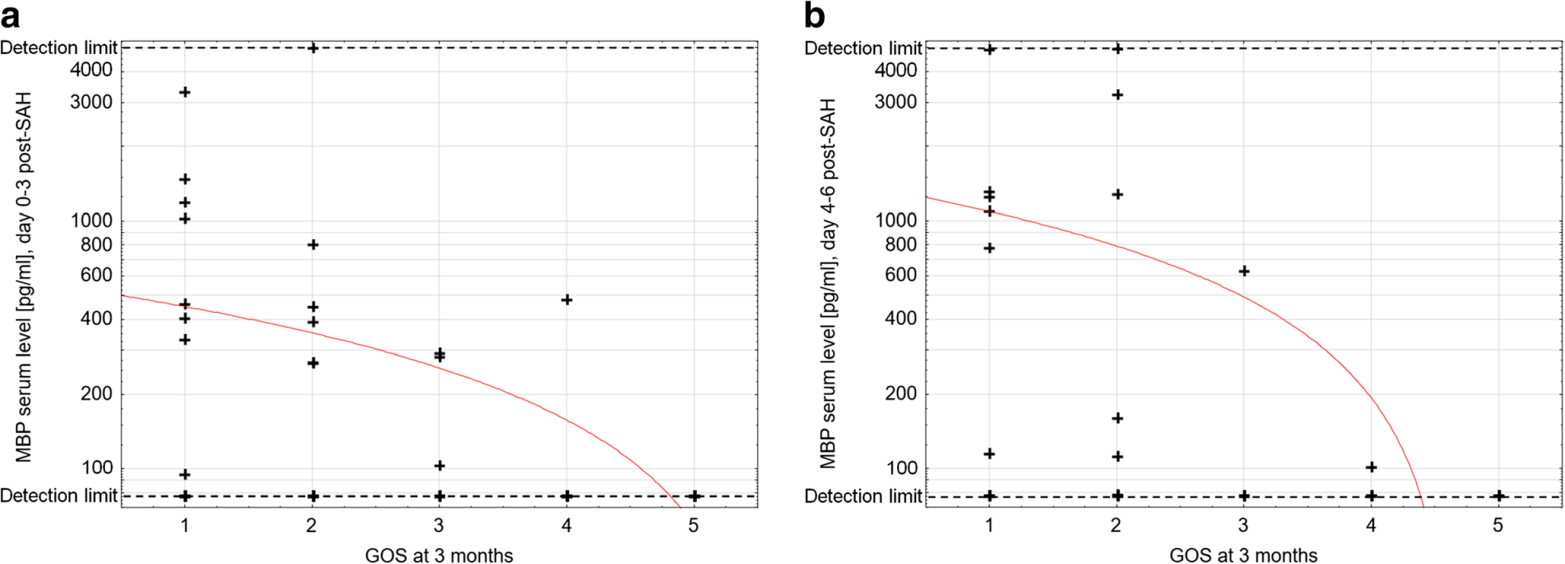
Scatter plot: Serum MBP level on days 0–3 and 4–6 post-SAH and treatment outcome. Spearman’s test was used. Dashed lines mark sensitivity thresholds of the ELISA kit. Serum MBP level is in logarithmic scale. **a** Correlation between MBP level on days 0–3 post-SAH and Glasgow Outcome Scale score at 3 months. Correlation coefficient = − 0.42. **b** Correlation between MBP level on days 4–6 post-SAH and Glasgow Outcome Scale score at 3 months. Correlation coefficient = − 0.45

Median MBP level on days 0–3 post-SAH was significantly increased in non-survivors (*p* = 0.005, Table 2). The Mann-Whitney *U* test also confirmed significantly higher MBP levels on days 0–3 post-SAH in unfavourable outcome patients (*p* < 0.001). Spearman’s test showed there was clinically relevant correlation between 3-month GOS and MBP level on days 0–3 and 4–6 post-SAH (cc = − 0.42 and cc = − 0.45, respectively; Table 3, Fig. 2). It is noteworthy that no patient with a GOS score of 5 (full recovery/low disability) had detectable levels of MBP in the early (days 0–3) samples. When considering the admission status, MBP levels approached but did not quite reach clinically relevant correlation with the three standard admission gradings (Glasgow Coma Scale, cc = − 0.37; Hunt and Hess grade, cc = 0.34; World Federation of Neurosurgical Societies scale, cc = 0.37).

**Table 2:** Two-group comparisons of MBP level on days 0–3, 4–6 and 9–12 post-SAH

	Group A	No. of patients in group A	Median MBP level (pg/ml)	Group B	No. of patients in group B	Median MBP level (pg/ml)	*p* value
Days 0–3 post-SAH	Non-survivors	22 (8)	744.0 (370–1335)	Survivors	102 (10)	344.1 (272–486)	*0.005*
	Poor outcome	52 (17)	406.5 (284–1025)	Favourable outcome	52 (1)	n/a	*< 0.001*
	Intracranial intervention	21 (13)	485.7 (333–1196)	No intracranial intervention	83 (5)	268.5 (104–393)	*< 0.001*
	DC	10 (9)	809.9 (333–1196)	EVD ± ICP bolt	11 (4)	430.9 (339–2728)	*0.020*
	ICH	23 (11)	462.8 (333–1025)	No ICH	81 (7)	284.2 (269–1475)	*< 0.001*
Days 4–6 post-SAH	Non-survivors	10 (6)	1173.4 (779–1318)	Survivors	22 (8)	394.2 (108–2272)	0.145
	Poor outcome	25 (13)	1096.0 (160–1318)	Favourable outcome	7 (1)	n/a	0.056
	Intracranial intervention	15 (10)	1173.4 (628–3255)	No intracranial intervention	17 (4)	137.6 (114–724)	*0.009*
	DC	7 (7)	1250.7 (628–3255)	EVD ± ICP bolt	8 (3)	779.5 (79–4951)	*0.029*
	ICH	10 (7)	1096.0 (628–3255)	No ICH	22 (7)	160.1 (113–1318)	*0.039*
Days 9–12 post-SAH	Non-survivors	16 (8)	1524.2 (773–3757)	Survivors	4 (2)	n/a	0.880
	Poor outcome	17 (9)	1769.5 (893–4593)	Favourable outcome	3 (1)	n/a	0.365
	Intracranial intervention	11 (6)	3553.3 (1770–5000)	No intracranial intervention	9 (4)	772.6 (366–1086)	0.273
	DC	3 (3)	1769.5 (458–2514)	EVD ± ICP bolt	8 (3)	5000 (4593–5000)	0.592
	ICH	7 (4)	2141.6 (114–3553)	No ICH	13 (6)	1085.8 (653–5000)	0.641

Mann-Whitney *U* test was used. Italicized text indicates a statistically significant difference with a *p* value less than 0.05. Medians and quartiles were calculated exclusively for patients with elevated MBP levels. Number of patients with elevated MBP level in each group is provided in brackets. In case of a group with less than 3 MPB-positive members, median MBP level was not calculated (in the table referred to as “n/a”)

*DC* decompressive craniectomy, *EVD* external ventricular drainage, *ICH* intracerebral haemorrhage, *ICP* intracranial pressure, *n* number of cases, *n/a* not available, *SAH* subarachnoid haemorrhage

**Table 3:** Correlation between MBP level and clinical data

	MBP level on days 0–3 post-SAH			MBP level on days 4–6 post-SAH			MBP level on days 9–12 post-SAH		
	*n*	cc	*p* value	*n*	cc	*p* value	*n*	cc	*p* value
GOS at 3 months	*104*	*− 0.42*	*< 0.001*	*32*	*− 0.45*	*0.010*	20	− 0.18	0.435
Age	104	0.21	0.034	32	0.28	0.127	20	0.10	0.687
HH grade on admission	104	0.34	< 0.001	32	0.30	0.100	20	0.22	0.357
WFNS grade on admission	104	0.37	< 0.001	32	0.10	0.598	20	− 0.02	0.944
GCS on admission	104	− 0.37	< 0.001	32	− 0.28	0.120	20	− 0.14	0.557
Modified Fisher score	104	0.27	0.005	32	0.15	0.409	20	0.25	0.297
Aneurysm size	104	0.27	0.006	32	0.40	0.023	20	0.36	0.122
ICH volume	*104*	*0.48*	*< 0.001*	*32*	*0.45*	*0.009*	20	0.23	0.337
CRP	90	0.30	0.004	*28*	*0.66*	*< 0.001*	18	0.38	0.120
WBC count	95	0.35	< 0.001	28	0.29	0.133	18	0.03	0.918
Hgb level	95	− 0.01	0.943	27	− 0.23	0.239	18	− 0.03	0.911
Glu level	67	0.13	0.280	23	− 0.19	0.395	13	− 0.35	0.240
Sodium	76	0.08	0.469	*24*	*0.47*	*0.021*	*13*	*0.87*	*< 0.001*
Potassium	74	− 0.04	0.757	24	0.29	0.168	13	0.33	0.264

Italicized text indicates a clinically relevant correlation with a correlation coefficient > 0.4 or < − 0.4. MBP level from each time point was correlated with laboratory results from corresponding day

*cc* correlation coefficient, *CRP* C-reactive protein, *HH* Hunt and Hess scale, *GCS* Glasgow Coma Scale, *Glu* glucose, *GOS* Glasgow Outcome Scale, *Hgb* haemoglobin, *ICH* intracerebral haemorrhage, *n* number of cases, *WBC* white blood cell, *WFNS* World Federation of Neurosurgical Societies scale

### Prior intracranial intervention

Intracranial interventions were carried out in 21 patients. These included intraparenchymal ICP monitor implantation, EVD implantation and DC with intracerebral haemorrhage (ICH) or subdural hematoma (SDH) evacuation where appropriate. In these patients, significantly higher levels of MBP were observed on days 0–3 and 4–6 post-SAH (*p* < 0.001 and *p* = 0.009, respectively; Table 2). Moreover, significantly higher levels of MBP were seen on days 0–3 and 4–6 post-SAH in those who underwent DC as opposed to those who had EVD and/or ICP bolt only (*p* = 0.020 and *p* = 0.029, respectively). In nine out of ten cases of DC, elevated MBP levels were observed early after intervention. In contradistinction, serum MBP was detectable in only four out of eleven patients with EVD and/or ICP monitor implanted.

### Intracerebral haemorrhage

**Fig. 3:**
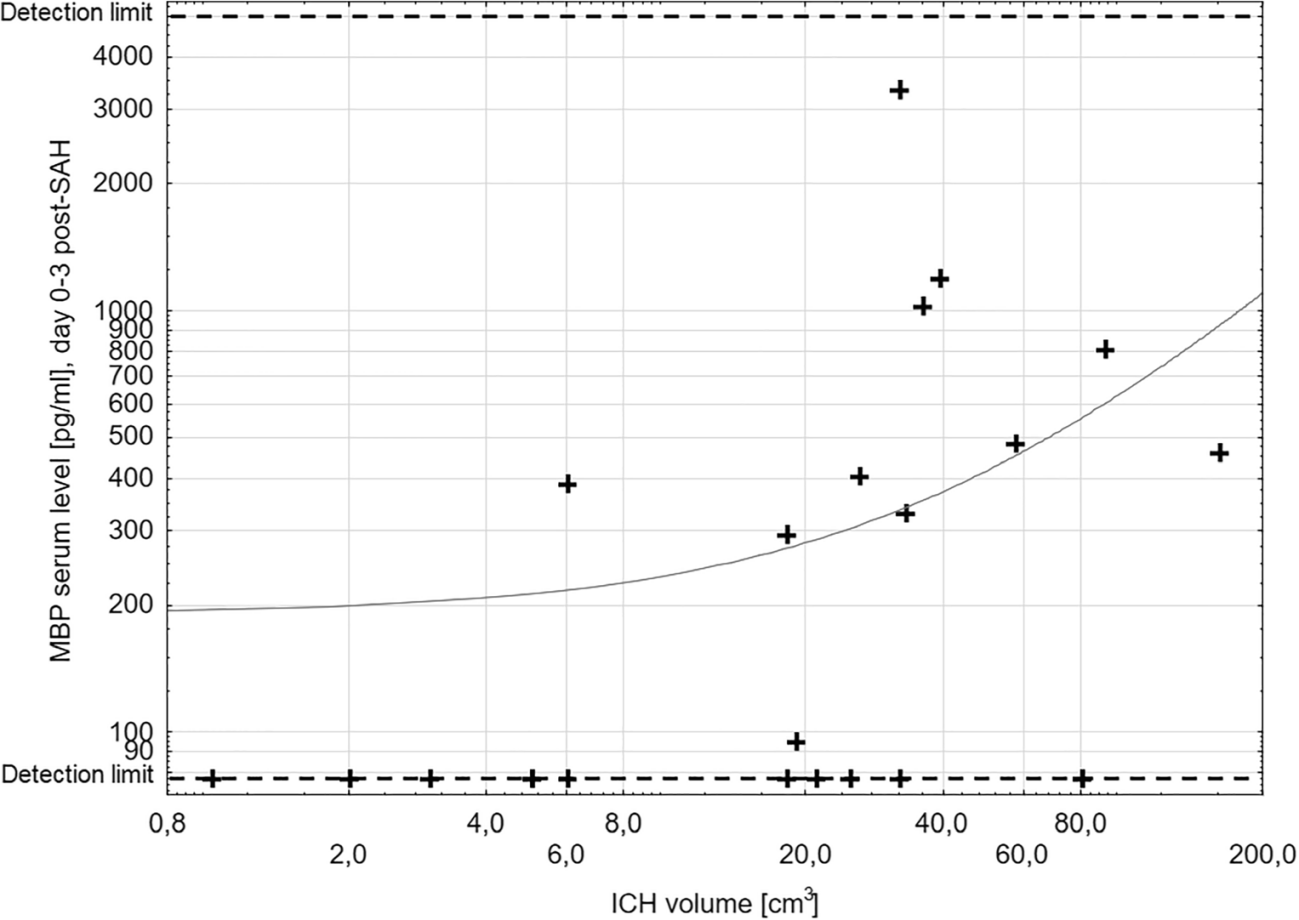
Scatter plot: MBP level on days 0–3 post-SAH and intracerebral haemorrhage volume. Dashed lines mark sensitivity thresholds of the ELISA kit. Both serum MBP level and ICH volume are in logarithmic scale. Spearman’s test was used. Correlation coefficient of 0.48 was found

ICH was found in 23 patients. The mean volume of the hematoma based on (a*b*c)/2 formula was 34.5 cm^3^. Previous intracranial intervention had been performed in 8 of patients with ICH; thus, the main cause of MBP elevation in these subjects cannot be established. Nevertheless, median MBP levels on days 0–3 and 4–6 post-SAH in patients with ICH are significantly elevated (*p* < 0.001 and *p* = 0.039, respectively; Table 2). To some extent, the early MBP level depends on the size of the hematoma, since Spearman’s test showed there was clinically relevant correlation (cc = 0.48) between MBP level on days 0–3 post-SAH and ICH volume (Table 3, Fig. 3).

### Demographic data, imaging findings and routine blood tests

At no stage was there any difference of MBP between the sexes; neither was there any correlation with the patients’ age or modified Fisher score. Among the six routine blood tests, the only significant correlations were for sodium on days 4–6 and 9–12 post-SAH (cc = 0.47 and cc = 0.87, respectively) and CRP on days 4–6 post-SAH (cc = 0.66).

## Discussion

The principal finding of this study is that the degree of parenchymal brain damage in SAH is reflected by the magnitude of the early MBP increase in peripheral blood. We have observed significantly higher levels of MBP on days 0–3 post-SAH in patients following surgical intervention and with large ICH compared with no intervention and small ICH/no ICH. The early MBP levels also correlated with the treatment outcome and with the ICH volume.

The role of MBP as a marker of brain damage is well accepted in the literature, yet the number of publications regarding SAH is very limited, only three papers were identified [4, 5, 12]. Hirashima et al. investigated 28 patients and found a correlation between CSF MBP levels on days 4–9 post-SAH and clinical grade on admission, extent of SAH, the presence of cerebral infarction and outcome [4]. In patients with traumatic brain injury, significant MBP elevation in blood samples drawn within hours of the ictus predicts non-survivors as well as CT scan evidence of intracranial bleeding or diffuse axonal injury [2, 15].

Our study also supports the safety of endovascular intervention in SAH. In 1984, Thomas et al. showed that cranial surgery in contrast to spinal/peripheral nerve surgery led to significant MBP elevation in peripheral blood [12]. The same team followed the MBP course in seven patients with SAH after aneurysm clipping. They observed elevated preoperative MBP levels, a slight postoperative decrease (for 4 days), followed by a gradual rise reaching a peak MBP level on the tenth postoperative day [5]. We have found that 86 out of 104 patients (83%) who underwent endovascular treatment had no MBP elevation. All patients in our study with elevated MBP either had ICH/SDH and/or required intracranial intervention.

According to our local practice, patients who are in good clinical condition after endovascular aneurysm occlusion return to their referring hospital, whilst those that are seriously ill remain for monitoring and treatment in our neurointensive care unit. This inevitably leads to an overrepresentation of poor outcome patients on days 4–6 and 9–12 post-SAH. Nevertheless, analysis of the raw data shows that patients without elevated MBP on days 4–6 and 9–12 post-SAH more frequently survive and achieve favourable outcome than those with increased MBP on the later dates. In the previously mentioned study by Hirashima et al., delayed increase of MBP in CSF (on days 4–9 post-SAH) was attributed to the occurrence of delayed cerebral ischaemia (DCI). MBP has been suggested to first be released into the CSF prior to clearance to the blood, and we suspect a similar relationship between DCI and serum MBP [2]. Unfortunately, follow-up data regarding infarction on head CT is incomplete, but in eleven patients sampled for MBP on days 9–12 post-SAH where follow-up scans are available, none of the MBP negative patients developed DCI, but all MBP-positive patients had evidence of DCI.

One surprising feature was the high correlation between sodium and MBP on days 4–6 and 9–12 post-SAH. This may be due to the relatively small number of subjects in this late phase, but may also be related to the use of hypertonic saline in the management of severely brain-injured patient, or to central diabetes insipidus in patients with massive brain injury.

There are several limitations in our study and other points that need to be addressed. First, we have noted a different range of MBP values in our study (up to 5 ng/ml), compared with previous, older publications (up to 100 ng/ml). This may be attributable to the different assay techniques; in the current study, ELISA was used, whereas previous studies used radioimmunoassay. Furthermore, the reference serum MBP in healthy adults has not been well established (ranges from < 30 to 60 pg/ml based on ELISA studies) [2, 9, 11]. Because of the relatively high sensitivity threshold of our ELISA kit (78 pg/ml), small elevations of MBP remained undetected. Second, only a small number of patients remained for sampling at days 4–6 and 9–12 post-SAH. Consequently, we considered it inappropriate to analyse over consecutive time points, since this would have meant neglecting the majority of samples collected on days 0–3 post-SAH. Third, following on from this, the more severely affected population is overrepresented; this limits our ability to draw conclusions about the role of MBP in the later stages following SAH. Fourth, the lack of a head CT follow-up schedule precludes appropriate analysis of the relationship between serum MBP levels and DCI occurrence [13]. Fifth, analysis of MBP levels would ideally be combined with analysis of well-established markers of brain injury in SAH, such as S100B protein or neuron-specific enolase [7, 10, 14]. Sixth, the current lack of rapid assays for MBP limits its clinical utility in everyday practice. We suggest that future studies should recruit full spectrum of SAH patients, follow rigorous blood and CSF sampling routine (including late SAH phase) and investigate relations between MBP, DCI and well-established markers of brain injury in SAH.

## Conclusion

Concentration of MBP in peripheral blood after intracranial aneurysm rupture reflects the severity of the brain parenchymal damage (due to surgery or ICH) and correlates with the treatment outcome. Endovascular aneurysm occlusion was not followed by a rise in MBP in most cases, suggesting the safety of this technique.

## Abbreviations

cc: Correlation coefficient
CNS: Central nervous system
CRP: C-reactive protein
CSF: Cerebrospinal fluid
CT: Computerised tomography
DC: Decompressive craniectomy
ELISA: Enzyme-linked immunosorbent assay
EVD: External ventricular drainage
GCS: Glasgow Coma Scale
Glu: Glucose
GOS: Glasgow Outcome Scale
Hgb: Haemoglobin
HH: Hunt and Hess scale
ICH: Intracerebral haemorrhage
ICP: Intracranial pressure
MBP: Myelin basic protein
SAH: Subarachnoid haemorrhage
SDH: Subdural hematoma
WBC: White blood cell
WFNS: World Federation of Neurosurgical Societies scale
